# Bacterial Suppression of RNA Polymerase II-Dependent Host Gene Expression

**DOI:** 10.3390/pathogens5030049

**Published:** 2016-07-13

**Authors:** Ines Ambite, Nataliya Lutay, Christoph Stork, Ulrich Dobrindt, Björn Wullt, Catharina Svanborg

**Affiliations:** 1 Department of Microbiology, Immunology and Glycobiology, Institute of Laboratory Medicine, Lund University, 22184 Lund, Sweden; nataliya.lutay@gmail.com (N.L.); bjorn.wullt@med.lu.se (B.W.); catharina.svanborg@med.lu.se (C.S.); 2 Institute of Hygiene, University Hospital Münster, Westfälische Wilhelms-Universität, 48149 Münster, Germany; christoph.stork@ukmuenster.de (C.S.); dobrindt@uni-muenster.de (U.D.)

**Keywords:** asymptomatic bacteriuria, RNA polymerase II, gene expression, transcriptional modulation, phages

## Abstract

Asymptomatic bacteriuria (ABU) is a bacterial carrier state in the urinary tract that resembles commensalism at other mucosal sites. ABU strains often lack the virulence factors that characterize uropathogenic *Escherichia coli* (*E. coli*) strains and therefore elicit weak innate immune responses in the urinary tract. In addition, ABU strains are active modifiers of the host environment, which they influence by suppressing RNA polymerase II (Pol II)-dependent host gene expression. In patients inoculated with the ABU strain *E. coli* 83972, gene expression was markedly reduced after 24 h (>60% of all regulated genes). Specific repressors and activators of Pol II-dependent transcription were modified, and Pol II Serine 2 phosphorylation was significantly inhibited, indicating reduced activity of the polymerase. This active inhibition included disease–associated innate immune response pathways, defined by TLR4, IRF-3 and IRF-7, suggesting that ABU strains persist in human hosts by active suppression of the antibacterial defense. In a search for the mechanism of inhibition, we compared the whole genome sequences of *E. coli* 83972 and the uropathogenic strain *E. coli* CFT073. In addition to the known loss of virulence genes, we observed that the ABU strain has acquired several phages and identified the lytic Prophage 3 as a candidate Pol II inhibitor. Intact phage particles were released by ABU during in vitro growth in human urine. To address if Prophage 3 affects Pol II activity, we constructed a Prophage 3 negative deletion mutant in *E. coli* 83972 and compared the effect on Pol II phosphorylation between the mutant and the *E. coli* 83972 wild type (WT) strains. No difference was detected, suggesting that the Pol II inhibitor is not encoded by the phage. The review summarizes the evidence that the ABU strain *E. coli* 83972 modifies host gene expression by inhibition of Pol II phosphorylation, and discusses the ability of ABU strains to actively create an environment that enhances their persistence.

## 1. Introduction

RNA polymerase II (Pol II) controls eukaryotic gene expression through the transcription of mRNA precursors, most snRNAs and microRNAs [1]. The assembly and control of the Pol II complex and the transcription cycle has been extensively characterized [2,3] but until recently, targeting of the host Pol II transcription machinery by bacteria has not been considered. Using gene expression analysis to screen patients with asymptomatic bacteriuria (ABU) for effects on gene expression, we discovered a marked effect on the human Pol II transcription machinery. The results identified Pol II suppression as a mechanism of bacterial adaptation to the urinary tract and a mechanism for ABU strains to actively modify the host environment in their favor.

In ABU, bacteria establish a “commensal-like” state, which might be regarded as “symbiotic”, as both bacteria and host benefit from this arrangement. In a placebo controlled longitudinal study, carriage of *E. coli* 83972 was shown to reduce the risk for symptomatic urinary tract infection (UTI) in patients with ABU compared to the placebo group and to delay the time to recurrence in the few patients, who developed symptoms [4,5,6,7]. As untreated acute pyelonephritis (APN) has lethal consequences, especially during pregnancy, these short term and long-term benefits of asymptomatic carriage may offer a significant evolutionary advantage.

The benefits of asymptomatic carriage for the bacteria have not been discussed, to the same extent, but may seem obvious. During ABU the bacteria thrive in an ecological niche with poor nutrient access but virtually no competition and weak antibacterial host defense in part due to a lack of virulence [4,8]. As a consequence, ABU strains persist for long periods of time, usually as a monoculture. Through Pol II inhibition, the innate immune response is reduced and there is little antibacterial activity to threaten bacterial survival. Based on the attenuation of virulence genes in *E. coli* 83972, we have proposed that ABU strains evolve towards commensalism and further attenuation has been demonstrated during growth in individual human hosts, after deliberate inoculation with *E. coli* 83972. These findings are consistent with a symbiotic relationship between bacteria and host.

The adaptive quality of asymptomatic carrier strains has generally been attributed to their lack of virulence [9]. They fail to trigger the disease associated, TLR4-dependent signalling pathways and persist in the host without overt inflammation and pathology. Genome sequencing and virulence genotyping has revealed that at least 50% of ABU strains have evolved from uropathogenic strains by inactivating mutations and deletions of virulence genes [10,11]. A reduction in total genome size compared to the virulent strains further suggested that ABU strains undergo reductive evolution to adapt to the host environment [12,13].

This review discusses the state of mutual “unresponsiveness” that characterises ABU [14]. In addition, we include new experimental data addressing the hypothesis that Prophage 3 in *E. coli* 83972 acts as a bacterial Pol II inhibitor in human cells.

## 2. Suppression of RNA Pol II-Dependent Gene Expression

The effect of ABU on host gene expression was studied in patients with recurrent UTI, who had undergone therapeutic bladder inoculation with the ABU strain *E. coli* 83972. Therapeutic efficacy of *E. coli* 83972 inoculation has been documented in placebo-controlled clinical trials and carriage of *E. coli* 83972 is safe [5,6,7]. After intravesical inoculation, the strain establishes long-term bacteriuria, which protects patients against super-infection with virulent strains [7].

**Figure 1:**
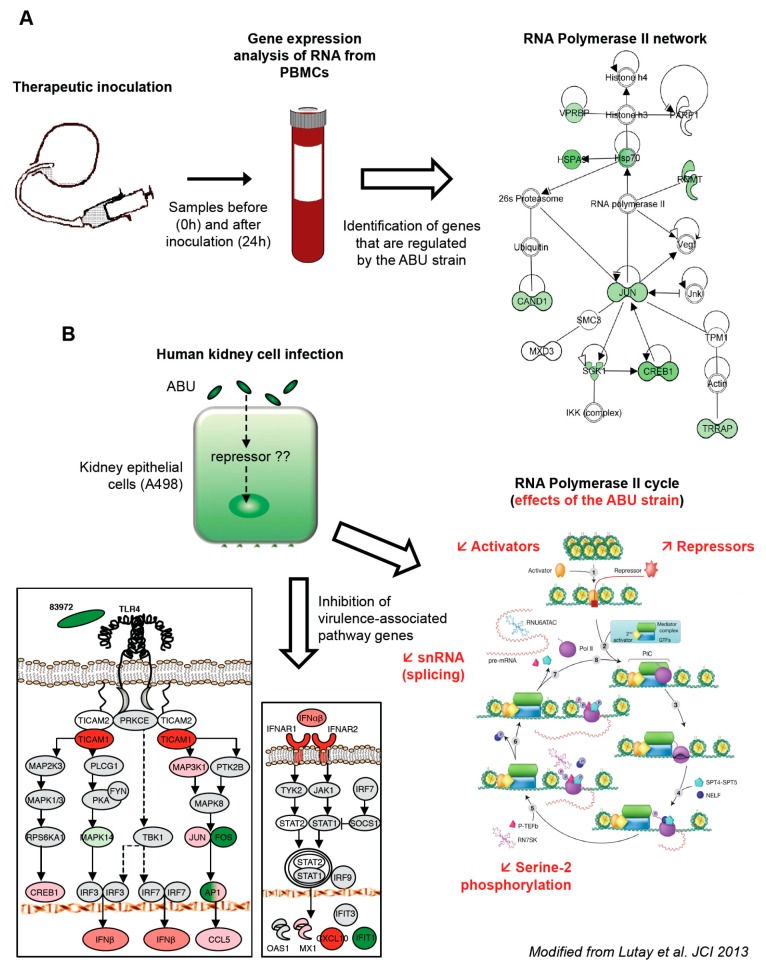
Suppression of gene expression in inoculated patients and infected human kidney cells. (**A**) Schematic of therapeutic inoculation with the ABU strain *E. coli* 83972, which established ABU in the inoculated patients. RNA was extracted from peripheral blood mononuclear cells (PBMCs) before and 24 h after intravesical inoculation. Significantly regulated genes were identified by transcriptomic analysis. Genes in the Pol II network were suppressed, by the ABU strain; (**B**) Human kidney epithelial cells (A498) were infected with the ABU strain *E. coli* 83972 and significantly regulated genes were identified by transcriptomic analysis, compared to uninfected cells. Pol II transcription cycle with gene categories regulated by ABU indicated in red. ABU infection affected different steps in the Pol II cycle, including chromatin opening, escape from pausing and pre-mRNA splicing. The ABU strain failed to activate/inhibit the pathology-associated TLR4 and IFN-β pathways. Adapted from Lutay et al. [14].

This therapeutic inoculation model has made it possible to evaluate how asymptomatic bacterial carriage affects the host. Blood samples were obtained at predetermined time points post inoculation (from 3 h to 4 weeks). To obtain a broad overview of the host response, peripheral blood RNA was harvested from the patients before and 24 h after inoculation with *E. coli* 83972 and significantly regulated genes were identified as fold change in gene expression (Figure 1A).

We first made the unexpected observation that the majority of genes were down-regulated in response to *E. coli* 83972, compared to the pre-inoculation sample in each host. A further analysis of significantly regulated genes identified effects on transcriptional regulation, including genes involved in DNA organization, innate immunity and inflammation. The “common” genes, which were regulated in all the inoculated patients, were further analyzed for network interactions. Based on established molecular pathways, a network of genes directly interacting with Pol II was identified (Figure 1A). All of the regulated genes in this network were suppressed, suggesting a direct effect of the ABU strain on Pol II-dependent transcription.

The regulation of Pol II dependent gene expression by *E. coli* 83972 was further analyzed in vitro, using human kidney carcinoma cells (A498). The responses to *E. coli* 83972 [5,15] and the uropathogenic *E. coli* (UPEC) strain CFT073 [16] were compared, after 4 h of infection. In addition to shared genes that were regulated by both strains, we identified a smaller group of genes that was exclusively regulated by the ABU strain. These included transcriptional repressors and activators, zinc-finger proteins, and regulators of translation. Other specifically regulated genes were involved in innate immune- and inflammatory responses, lipid metabolism, protein transport, ubiquitin cycle, G protein signaling pathway and Rho-Ras signaling [14]. In addition to the modulation of a broad range of transcription activators and repressors associated with Pol II (Figure 1B), the overall Pol II-dependent gene expression was markedly reduced in ABU infected cells.

The mucosal innate immune response is triggered by bacterial virulence ligands and their interactions with receptors on epithelial cells or other mucosal cell types. For example, the binding of P fimbriae to glycosphingolipid receptors activates the pathogen specific TLR4/CREB/IRF3/7 pathway [17,18]. UPEC also stimulate the expression of IFN-β, which is essential to establish a functional antibacterial defense in the kidneys [18]. The expression of *TLR4*, the kinase *MAP3K1*, the transcription factors *JUN* and *CREB1*, and the cytokines *CCL5* and *IFNB1* was low in ABU infected cells (Figure 1B). Moreover, the ABU strain suppressed *MAPK14* and *FOS*, downstream of TLR4, and *IFIT1*, downstream of IFN-β. The results confirmed and extended the results of the human inoculation study, suggesting that virulence pathway and the Pol II transcription cycle is inhibited in hosts that carry ABU strain.

## 3. Inhibition of RNA Polymerase II by ABU Strains

**Figure 2:**
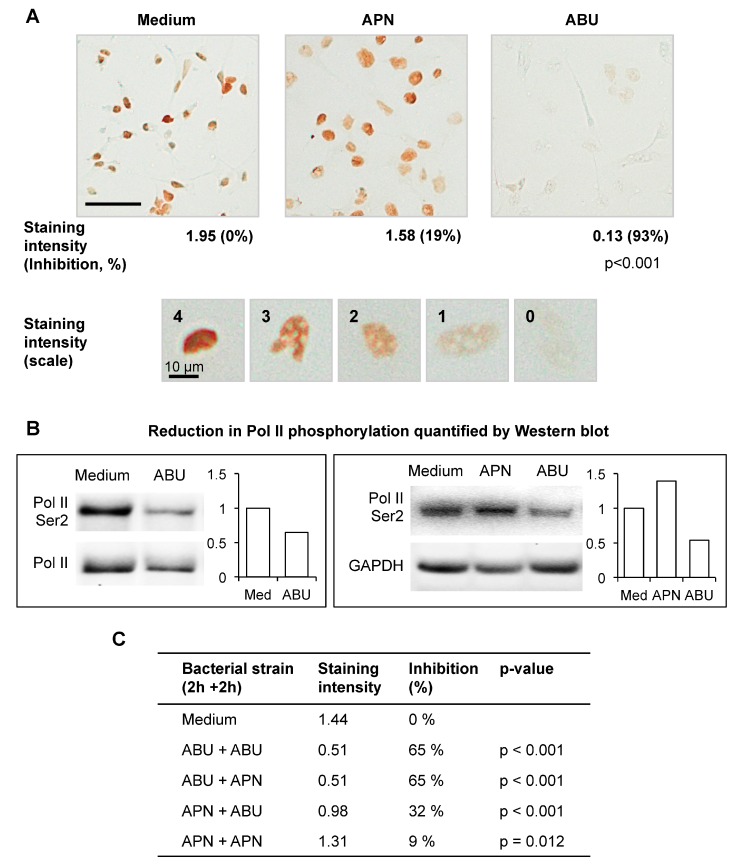
The ABU strain inhibits Pol II Ser 2 phosphorylation in vitro. (**A**) Immunoperoxidase staining of human kidney epithelial cells (A498), using HRP-conjugated antibodies. Pol II phosphorylation (brown) was reduced by the ABU strain compared to uninfected cells. A scale representing each staining intensity is shown (4 = brown, 0 = colorless). Inhibition of Pol II phosphorylation was expressed in percent of the uninfected controls (0% = control, no inhibition; 100% = complete inhibition). The cells were exposed to 2 × 10^9^ CFU/mL of *E. coli* CFT073 (APN) or 83972 (ABU), (*n* = 100 cells per sample, *p*-value by χ^2^ test). Scale bar = 50 μm; (**B**) Phospho-Pol II in human kidney epithelial cells, infected with the ABU or APN strains. Western blots of whole cell extracts. The phospho-specific staining was normalized against total Pol II or GAPDH; (**C**) Competitive infection. The strain to which the cells were first exposed, was shown to determine Pol II activation. Pol II Ser 2 staining of human kidney epithelial cells after 2 h + 2 h infection with the ABU strain followed by the APN strain or the APN strain followed by the ABU strain (*n* ≥ 167 cells, *p*-values by χ^2^ test).

The productive mRNA elongation step is generally marked by the phosphorylation of Serine 2 (Ser 2) residues in the Pol II carboxy-terminal domain, by P-TEFb (the kinase complex, positive transcription elongation factor b) [3]. Ser 2 phosphorylation of Pol II is therefore used as an indicator of Pol II activity. The A498 human kidney epithelial cell line was infected with *E. coli* 83972 (ABU) or CFT073 (APN), for 4 h and Pol II Ser 2 phosphorylation was quantified by immunoperoxidase staining (Figure 2A) and western blots (Figure 2B), using specific antibodies. The ABU strain was shown to inhibit Pol II Ser 2 phosphorylation in a concentration dependent manner, compared to uninfected or APN-infected cells (93% and 19% inhibition, respectively, compared to uninfected control).

To address if exposure of the cells to a virulent strain affects the Pol II response to the ABU strain, human kidney cells were successively infected with the ABU strain (2 h) and the APN strain (2 h) (Figure 2C). The response was then analyzed by Pol II Ser 2 staining and Western blot. The APN strain did not modify the response to the ABU strain when added after the first incubation period with the ABU strain (65% inhibition for the two conditions). Furthermore, the ABU strain was able to reduce Pol II phosphorylation in APN infected cells (from 9% to 32% inhibition). These observations suggest that the propensity to inhibit Pol II and modify the host environment is a characteristic of the ABU strain and is not counteracted by APN infection.

To determine if Pol II inhibition is a common feature among ABU strains, we screened a collection of ABU strains using the anti-Pol II-Ser 2 antibody. The frequency of the Pol II inhibitory phenotype was determined in a collection of epidemiologically defined ABU isolates (*n* = 75) from which *E. coli* 83972 was derived [19]. The ABU strains were compared to acute pyelonephritis strains (*n* = 88), obtained from children, who were admitted with febrile UTI to the children’s hospital in the same area. Pol II activation was quantified by immunoperoxidase staining after in vitro infection with each strain and classified depending on the degree of inhibitory activity. In total, 37% of the ABU strains showed strong inhibitory activity compared to 17% in the APN group and 80% were inhibitory [14]. The results suggest that inhibition of Pol II activation is a common feature of asymptomatic carrier strains.

## 4. Prophage 3 as a Putative RNA Pol II Inhibitor

To identify candidate genes responsible for the inhibition of Pol II, we compared the *E. coli* 83972 genome sequence (GenBank CP001671 [4]) to that of *E. coli* CFT073 (GenBank AE014075.1 [20]), which did not significantly reduce Pol II phosphorylation (Figure 2A). *E. coli* CFT073 and *E. coli* 83972 share genomic regions encoding virulence and fitness-associated factors, for example iron-uptake systems, fimbriae and adhesins, toxins, capsules and secretion systems [4,12].

*E. coli* 83972 was shown to have acquired 6 prophages, including the lytic Prophage 3 (1363465–1413287), which encodes the *sit* genes involved in the iron/manganese uptake system. Prophage 1 (361604–387673) showed sequence homology to *E. coli* 042, an entero-aggregative *E. coli* strain that causes diarrhea [21]. Prophage 2 (1193402–1208354) showed homology to certain enterohaemorrhagic *E. coli* prophages and was inserted into the *focD* gene, thus disrupting F1C fimbrial expression. Prophage 4 (1804935–1842196 in CP001671) had sequence-similarities to prophages found in the genomes of one UPEC strain (GenBank CU928164.2) and two *Salmonella enterica* serovar Typhi strains (GenBank AE014613.1 and AL513382.1).

**Figure 3:**
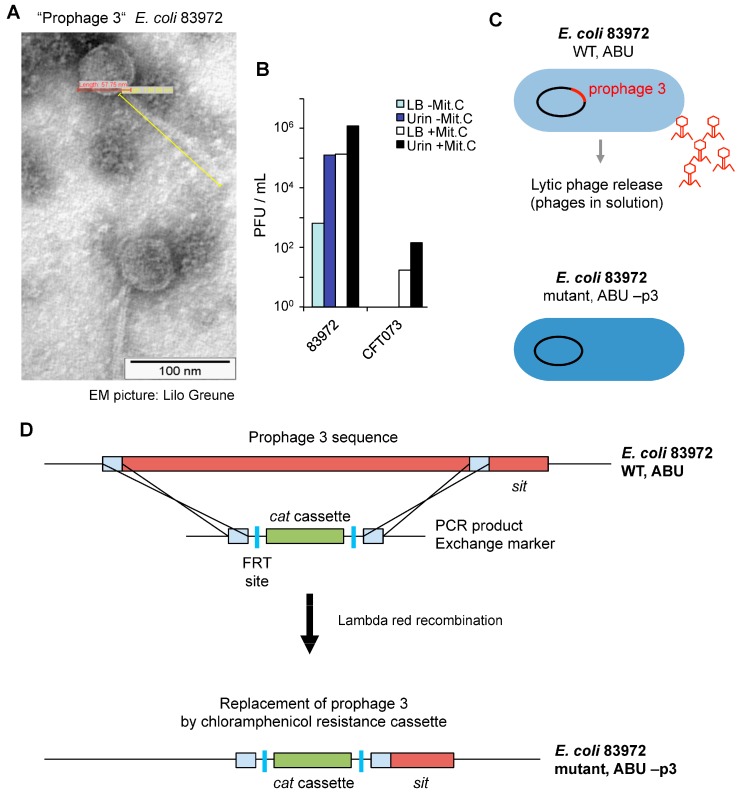
Induction of Prophage 3 by *E. coli* 83972 and construction of the *E. coli* 83972 Prophage 3 deletion mutant. (**A**) Electron microscopy of lambda-like Prophage 3 released by *E. coli* 83972 during in vitro growth in pooled human urine; (**B**) Quantification of lytic phages by plaque formation (plaque forming units, PFU) on *E. coli* C600. *E. coli* 83972 released lytic phages during growth in urine and Luria Bertani (LB) broth. Moderate release of lytic phages by CFT073 was induced by mytomicin C; (**C**) The Prophage 3 sequence was deleted from the *E. coli* 83972 chromosome. The ABU—p3 mutant strain is unable to produce and release lytic Prophage 3 particles; (**D**) The Prophage 3 sequence was replaced by a chloramphenicol acetyltransferase (*cat*) cassette, using homologous Lambda red recombination technology and chloramphenicol resistance for selection. The bacterial *sit* gene, involved in iron uptake, was conserved in the mutant strain. In the construct, the chloramphenicol resistance gene is surrounded by two flippase recognition target (FRT) sites.

Phage particles were detected by electron microscopy imaging, after in vitro growth of *E. coli* 83972 in pooled sterile human urine. The phages showed lambda-like morphology and were 58 nm in diameter and with a 137 nm tail. The phages were not detected after growth of *E. coli* CFT073 under the same conditions (Figure 3A). The lytic ability of *E. coli* 83972 Prophage 3 was assessed by growth inhibition of *E. coli* C600 and quantified as plaque forming units (PFU) (Figure 3B). During growth in urine, *E. coli* 83972 produced more PFU than during growth in complete Luria Bertani (LB) broth (10^5^ versus 657 PFU/mL). Treatment with mitomycin C increased the PFU formation by *E. coli* 83972 grown in urine or broth as well as the release of lytic phages from *E. coli* CFT073 (17.5–142 PFU/mL, Figure 3B). Mitomycin C, a potent DNA crosslinker, induces the release of prophages by bacteria.

To address if Prophage 3 is involved in the mechanism of Pol II inhibition, Prophage 3 was deleted from the chromosome of *E. coli* 83972 (Figure 3C). Lambda red-mediated recombineering technology [22] was used to replace, with a chloramphenicol resistance cassette (*cat*), the a 44-kb chromosomal region that contains the Prophage 3 genome sequence. The bacterial *sit* gene was conserved in the mutant strain. In the construct, two flippase recognition target (FRT) sites surrounded the *cat* gene (Figure 3D).

**Figure 4:**
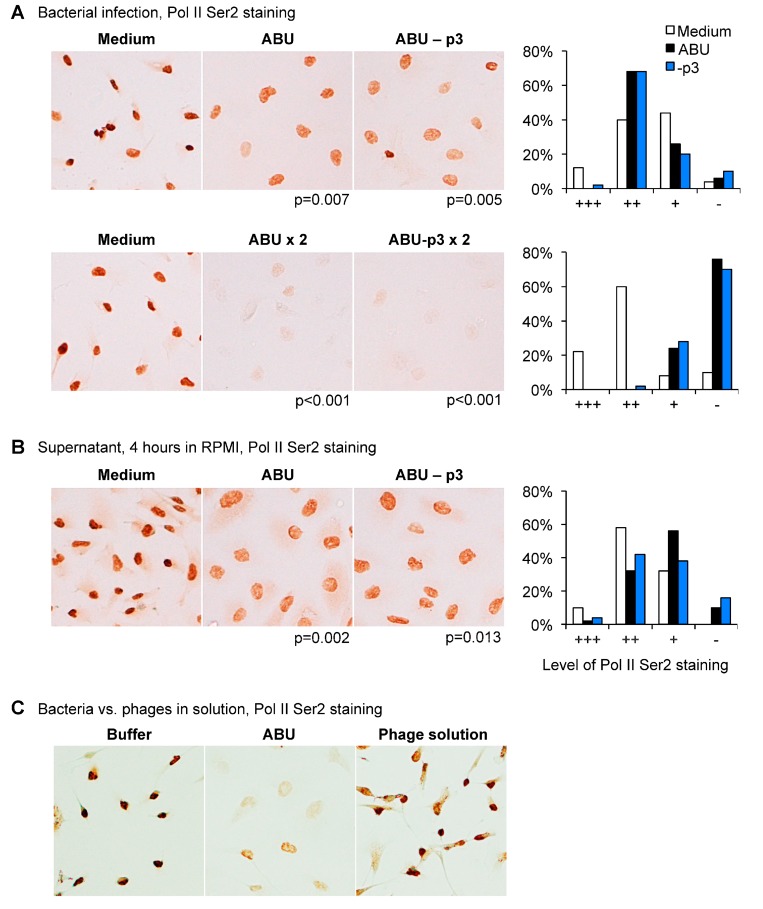
Pol II phosphorylation is not altered by the Prophage 3 deletion. (**A**) The Pol II Ser 2 staining intensity (brown) did not differ between cells infected with the ABU strain or the -Prophage 3 deletion mutant (10^9^ and 2 × 10^9^ CFU/mL, *n* = 100 cells, *p*-values by χ^2^ test). Histograms show the distribution of human kidney epithelial cells according to their Pol II Ser 2 staining intensity (+++ = highly stained, - = no staining); (**B**) Supernatants of A498 cells infected with the ABU strain or the ABU-Prophage 3 deletion mutant. Similar effects on Pol II phosphorylation (10^9^ CFU/mL, *n* = 100 cells, *p*-values by χ^2^ test). Pol II Ser 2 staining was quantified by immunoperoxidase staining using Pol II Ser2specific antibodies; (**C**) Pol II Ser 2 phosphorylation was not inhibited by lytic phage particles released by the ABU strain during growth in human urine, in vitro.

Human kidney cells (A498) were subsequently infected with *E. coli* 83972 (ABU+p3) or the Prophage 3 negative mutant (ABU-p3) and RNA Pol II phosphorylation was compared. No difference was observed between the two strains, both wild type (WT) and mutant inhibited Pol II Ser2 phosphorylation in a dose-dependent manner (Figure 4A). The supernatant of the Prophage 3 negative mutant did not suppress Pol II phosphorylation (Figure 4B).

These results were confirmed using a suspension of phages released by the ABU strain. The phage mixture did not inhibit Pol II Ser 2 phosphorylation in human kidney epithelial cells (Figure 4C). The results suggest that the functions of Prophage 3 are unrelated to RNA Pol II phosphorylation in host cells.

## 5. Discussion

Deliberate inoculation with *E. coli* 83972 is used as an alternative to antibiotic therapy in patients with recurrent UTI [7,23,24,25]. In placebo-controlled studies, asymptomatic carriage of this strain has been shown to protect the patient against super-infection with more virulent strains and to reduce the number of symptomatic UTI episodes. In addition to the protection against symptomatic UTI, the patients experience a sense of well being, that is lost if they spontaneously clear the strain. It is therefore important to understand the molecular mechanisms that define the crosstalk between bacteria and host. As discussed in this review, the bacteria inhibit host gene expression through an effect on RNA Pol II, providing a molecular mechanism for host unresponsiveness and especially the inhibition of the innate immunity and inflammation. Based on the attenuation of innate immunity in inoculated patients, targeting of RNA Pol II may be useful as a new tool to modify inflammation in susceptible hosts.

ABU strains establish a form of commensalism that allows bacteria to expand their population size and persist at advantageous sites in the host. Contrary to the prevailing dogma that virulence attenuation renders the host inert to the ABU strains, we show that these strains attenuate the antibacterial effector functions of the host by suppressing gene expression. We have shown that *E. coli* strains from asymptomatic carriers actively repress the RNA polymerase II transcriptional machinery. The majority of ABU strains from a population-based screen shared the ability to suppress Pol II activation (Ser 2 phosphorylation), suggesting that this active molecular solution has evolved in parallel with the loss of virulence in the ABU strains.

The broad effect against RNA Pol II-dependent transcription includes genes in the innate immune system that are activated by UPEC strains. These include transcription factors Fos and Jun, which form the AP1 transcription complex, as well as genes encoding key inflammatory mediators. The broad effects also suggest that a variety of other host cell functions are being affected. These effects remain to be studied further.

The bacterial mechanism of Pol II inhibition has not been identified. In this study, we examined the genome of *E. coli* 83972 to identify genes that were not present in the chromosome of UPEC strains like CFT073. Several phage genomes were detected. Phages are important mobile elements for the transfer of genes between bacteria, to promote adaptation to new host environments. Phages have been identified as carriers of virulence factors in pathogens, such as *Shigella dysenteriae* (Shiga toxin genes) or enterotoxigenic *E. coli* strains [26,27]. In addition, bacteriophages have been extensively studied as antimicrobial agents [28]. We therefore addressed if phages, which have been acquired by *E. coli* 83972, might be responsible for Pol II suppression. The ABU strain *E. coli* 83972 was found to release phage particles during growth in human urine, and we selected Prophage 3 as a candidate regulator of host gene expression. Using a phage deletion mutant, we found no evidence in support of phage-mediated regulation of Pol II activity. The identification of phage DNA in the genome of *E. coli* 83972 suggested that phages might serve as carriers of information to promote bacterial adaptation to the human urinary tract. The induction during growth in urine strongly suggests a role in bacterial adaptation, which remains to be identified.

## 6. Conclusions

During asymptomatic carriage, ABU strains actively control the host environment by manipulating gene expression. The ABU strain *E. coli* 83972 inhibits RNA Polymerase II-dependent gene expression and the expression of innate immune response genes. By unraveling this new mechanism of transcriptional modulation, we shed light on ABU as a potentially symbiotic state that benefits the bacteria and can be established therapeutically, for prevention of recurrent urinary tract infection.
